# Temporal Trends and Prognostic Impact of Pacemaker-Associated Heart Failure: Insights from a Nationwide Cohort Study

**DOI:** 10.3390/jcm14217744

**Published:** 2025-10-31

**Authors:** Young Jun Park, Sungjoo Lee, Sungjun Hong, Kyunga Kim, Juwon Kim, Ju Youn Kim, Kyoung-Min Park, Young Keun On, Seung-Jung Park

**Affiliations:** 1 Division of Cardiology, Department of Internal Medicine, Wonju Severance Christian Hospital, Yonsei University Wonju College of Medicine, Wonju 26426, Republic of Korea; pyj@yonsei.ac.kr; 2 Department of Digital Health, Samsung Advanced Institute for Health Sciences & Technology, Sungkyunkwan University, Seoul 03036, Republic of Korea; danhalee2016@gmail.com (S.L.); hsj2864@gmail.com (S.H.); 3 Biomedical Statistics Center, Research Institute for Future Medicine, Samsung Medical Center, Seoul 06351, Republic of Korea; 4 Division of Cardiology, Department of Internal Medicine, Heart Vascular and Stroke Institute, Samsung Medical Center, Sungkyunkwan University School of Medicine, Seoul 06351, Republic of Korea; abcd186a@gmail.com (J.K.); kzzoo921@gmail.com (J.Y.K.); bkm1101@hanmail.net (K.-M.P.); oykmd123@gmail.com (Y.K.O.)

**Keywords:** pacemaker, heart failure, mortality, predictor, nationwide registry

## Abstract

**Background/Objectives:** Pacemaker-associated heart failure (PaHF) is a recognized complication of chronic ventricular pacing, yet its long-term incidence and prognostic impact remain incompletely defined. Previous studies on PaHF have been largely limited by small sample sizes, single-center designs, and insufficient long-term or time-dependent analyses. We aimed to evaluate the incidence, clinical predictors, and mortality risk of PaHF in a nationwide real-world cohort. **Methods:** Using the Korean National Health Insurance Service database, we identified 32,216 patients who underwent de novo pacemaker implantation between 2008 and 2019 without prior heart failure. **Results:** During a median follow-up of 3.8 years, 4170 patients (12.9%) developed new-onset PaHF and 6184 (19.2%) died. PaHF was independently associated with increased all-cause mortality (adjusted hazard ratio [HR]: 3.11, 95% confidence interval [CI]: 2.93–3.32, *p* < 0.001), even after accounting for immortal-time bias and relevant covariates. The incidence of PaHF and its associated mortality risk both peaked within the first six months post implantation and remained persistently elevated throughout follow-up; notably, PaHF-associated mortality showed a late resurgence. Sensitivity and subgroup analyses consistently demonstrated higher mortality among patients with PaHF across a wide range of clinical characteristics. **Conclusions:** In this large, nationwide cohort, the development of PaHF was associated with a substantial and sustained increase in mortality risk following pacemaker implantation. Given the persistent and dynamic nature of this risk, longitudinal monitoring of cardiac function and individualized pacing strategies may be warranted to mitigate long-term adverse outcomes. Additionally, these findings provide real-world benchmarks to guide future pacing strategies and surveillance efforts.

## 1. Introduction

Permanent pacemaker (PPM) implantation remains the cornerstone therapy for patients with symptomatic bradyarrhythmia, such as sinus node dysfunction (SND) or atrioventricular block (AVB). However, pacing-induced left ventricular (LV) electromechanical dyssynchrony can lead to progressive LV dilatation, LV ejection fraction (EF) deterioration, and, eventually, overt heart failure (HF), a condition commonly referred to as pacemaker-or pacing-associated HF (PaHF) [1,2,3].

Previous studies estimate that PaHF occurs in approximately 6% to 25% of patients within 2 to 4 years following PPM implantation [1,4,5,6]. Several risk factors have been implicated, including advanced age, male sex, pre-existing LV systolic dysfunction, baseline left bundle branch block, right ventricular (RV) apical pacing, prolonged paced QRS duration, and a higher RV-pacing burden [1,2,3,4,5,6,7]. Importantly, the onset of PaHF has been linked to a significant increased mortality compared to patients without HF following pacing [1,5], underscoring the need for a deeper understanding of this clinical entity.

Despite these findings, most of the current evidence is derived from single-center or limited-scale studies with modest sample sizes and relatively short follow-up durations. These constraints limit the ability to evaluate the long-term prognostic impact of PaHF in real-world settings. Furthermore, existing literature has largely focused on early-phase risk following PPM implantation, with limited data available on the long-term incidence of PaHF or the long-term mortality risk associated with its development over time. In addition, prior studies have seldom accounted for immortal time bias, which may have led to inaccurate estimation of the temporal association between PaHF and mortality [1,4,5].

To address these gaps, we conducted a nationwide population-based cohort study using a comprehensive claims database to (1) assess the incidence of PaHF over an extended follow-up period after de novo PPM implantation, (2) identify clinical factors associated with its development, and (3) determine the time-dependent prognostic impact of incident PaHF on long-term all-cause mortality, using appropriate time-varying models to account for immortal time bias.

## 2. Materials and Methods

### 2.1. Data Sources

This nationwide retrospective cohort study was conducted using the National Health Information Database (NHID), curated by the Korean National Health Insurance Service (NHIS)—a government-managed, single payer system that provides healthcare coverage for nearly the entire South Korean population (≥52 million individuals) [8]. The NHID integrates longitudinal health-related data, encompassing demographic information, diagnostic codes (based on the International Classification of Disease 10th Revision), inpatient and outpatient claims, medication prescription (coded using the Anatomical Therapeutic Chemical classification system), surgical and procedural records, use of medical devices, and death registry data.

The database was launched as a nationwide platform in 2000 and made available for research use through the NHIS data sharing service beginning on 10 September 2009. It has since served as a key source for public health and clinical research in Korea. The validity of diagnostic coding within the NHID has been previously validated for several major cardiovascular or rare diseases, including myocardial infarction (92% accuracy) and hypertrophic cardiomyopathy (93% accuracy), demonstrating high concordance with medical records [8,9,10,11].

### 2.2. Study Design and Population

This study cohort consisted of adult patients (aged ≥ 18 years) who underwent initial de novo PPM implantation between 1 January 2008 and 31 December 2019. Eligible cases were identified using NHIS claims codes for PPM procedure and devices (Supplementary Table S1).

Patients were excluded if they had a prior history of HF, defined as hospitalization with an HF diagnosis (ICD-10: I50.9) or use of angiotensin receptor–neprilysin inhibitor (ARNI) before the index implantation. Additional exclusions included patients undergoing reimplantation of pulse generators or leads following PPM system removal, or those who died on the day of PPM implantation.

### 2.3. Data Acquisition

Baseline demographic characteristics, including age and sex, were extracted from the claims data. Comorbid conditions were determined using ICD-10 diagnostic codes recorded from one year prior to the index date until the onset of PaHF (Supplementary Table S2). Overall comorbidity burden was quantified using the Charlson Comorbidity Index, derived from corresponding ICD-10 codes (Supplementary Table S3). Medication records were obtained with a focus on renin-angiotensin system (RAS) inhibitors including angiotensin-converting-enzyme inhibitors (ACEIs), angiotensin II receptor blockers (ARBs), and ARNI. Additional medication classes, including beta-blockers, mineralocorticoid receptor antagonists (MRAs), loop and thiazide diuretics, antiplatelet agents, and anticoagulants, were also identified from prescription records. Device-related variables, such as pacemaker type and subsequent cardiac resynchronization therapy (CRT)-upgrade, were identified using claim codes (Supplementary Table S1).

### 2.4. Study Outcomes and Follow-Up

The co-primary outcomes of this study were (1) development of PaHF and (2) all-cause mortality following de novo PPM implantation. PaHF was stringently defined as a new post-PPM hospitalization with an HF diagnosis (ICD-10: I50.9), accompanied by at least two prescriptions for guideline-directed medical therapy for HF including ACEIs/ARBs, beta-blockers, or MRAs. Additionally, patients who underwent a CRT-upgrade or initiated ARNI therapy post-implantation were also categorized as PaHF cases, even in the absences of formal HF diagnosis code or other HF medications.

Sensitivity analyses using a broader definition of PaHF (new HF diagnosis regardless of therapy) were also conducted. A detailed comparison of the strict and broad definitions is provided in Supplementary Table S4. All main analyses were conducted based on the strict PaHF definition except sensitivity analyses according to the broad PaHF definition. Time-to-PaHF was calculated from the date of PPM implantation to the date of first qualifying PaHF event, and follow-up was censored at the earliest occurrence of death, a competing non-pacing-related HF event (e.g., myocardial infarction, myocarditis, alcoholic cardiomyopathy, cardiac sarcoidosis, and cardiac amyloidosis; Supplementary Table S5), or the end of the study period (31 December 2019).

For mortality analysis, the follow-up period began at the date of PPM implantation and ended at either the date of death or last follow-up, whichever occurred earlier.

### 2.5. Statistical Analyses

Descriptive statistics were used to summarize baseline characteristics, with continuous variables expressed as mean ± standard deviation (SD) and categorical variables presented as counts with corresponding percentages. For group comparisons, the Student’s *t*-test or the Mann–Whitney U test was applied for continuous variables depending on distributional assumptions, and the chi-square or Fisher’s exact test was used for categorical variables, as appropriate.

The incidence rates of PaHF and all-cause mortality were expressed as events per 100 person-years (PY) with exact 95% confidence intervals (CIs) derived from Poisson distribution methods. Cumulative incidence functions for PaHF were plotted using the Kaplan–Meier methods, while instantaneous hazard rates were modeled using a nonparametric B-spline smoothing technique embedded in a generalized linear mixed model framework, capturing temporal dynamics throughout the follow-up period [12].

To identify risk factors associated with the onset of PaHF, multivariable Cox proportional hazards models were constructed, and adjusted hazard ratios (HRs) with 95% CIs were computed. To evaluate the prognostic impact of PaHF on mortality, PaHF was treated as a time-dependent exposure to address immortal-time bias, defined as the period between PPM implantation and PaHF diagnosis (Supplementary Figure S1). We applied an extended Kaplan–Meier survival analysis framework that dynamically reclassified patients at the time of PaHF diagnosis, allowing unbiased estimation of cumulative mortality [13,14]. Additionally, we utilized time-dependent Cox models incorporating cubic spline terms to flexibly evaluate how the timing of PaHF influenced mortality risk across the entire follow-up period [15].

To evaluate a possible modification on the association between the PaHF and mortality, we conducted subgroup analyses stratified by age (<65, 65–75, and >75 years), sex, comorbidities, pacemaker type (single- vs. dual-chamber), pacing indication (AVB vs. SND), and medication (user vs. non-user). The potential modification was tested by assessing the interaction term of each subgrouping variable with PaHF.

For multivariable model construction, covariates were selected based on both univariable significance (*p* < 0.05) and clinical relevance.

Multicollinearity was assessed by the variance inflation factor, which, at a value greater than 4, was considered to indicate non-negligible collinearity. All proportional hazards assumptions were verified by evaluating scaled Schoenfeld residuals. Adjusted HRs and their 95% CIs were reported for all time-to-event analyses. A two-tailed *p* value < 0.05 was considered statistically significant. Statistical computations were performed using SAS software version 9.3 (SAS Institute, Cary, NC, USA) and R software version 4.1.0 (The R Foundation for Statistical Computing).

## 3. Results

### 3.1. Baseline Characteristics and Incidence of PaHF

**Figure 1:**
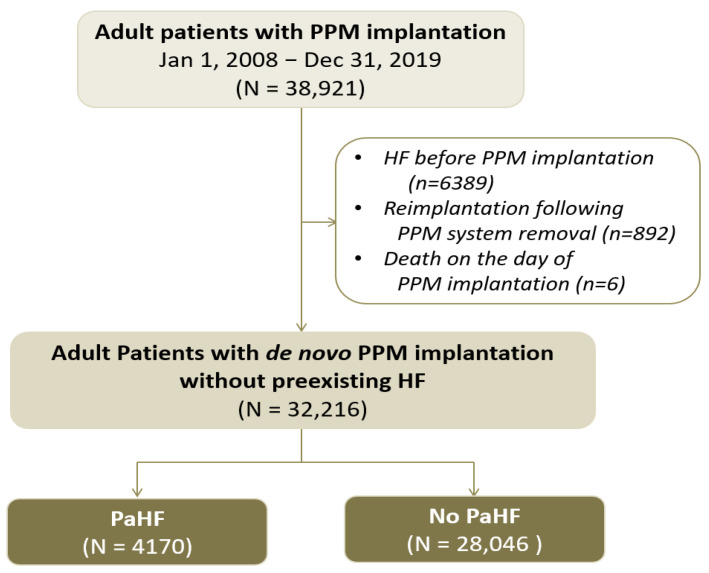
Study flow of the permanent pacemaker cohort.

As illustrated in Figure 1, a total of 38,921 adult patients underwent PPM implantation during the study period. After excluding patients with preexisting HF (*n* = 6389), reimplantation following device removal (*n* = 892), or death on the day of the procedure (*n* = 6), a final cohort of 32,216 patients with de novo PPM implantation and no history of HF was identified for analysis. The mean age was 70.6 ± 12.1 years. Among them, 13,632 (42.3%), 20,246 (63.4%), and 27,073 (84.0%) had male sex, AVB, and dual-chamber PPMs, respectively.

During the median follow-up of 3.8 (interquartile range, 1.7–6.7) years, 4170 of 32,216 patients (12.9%) developed new-onset PaHF during follow-up, with an incidence rate of 3.3 per 100 PYs (95% CI, 3.2–3.4) (Figure 1 and Figure S2). The mean time-to-PaHF was 3.0 ± 2.8 years. When applying a broader definition of PaHF (Supplementary Table S4), a total of 6118 patients (19.0%) were classified as PaHF, corresponding to a higher incidence rate of 4.9 per 100 PYs (95% CI, 4.8–5.0).

Baseline characteristics stratified by PaHF status are summarized in Table 1. Patients who developed PaHF exhibited substantially higher risk profiles for most clinical variables: more advanced age, a higher proportion of comorbidities, and more frequent use of medications.

### 3.2. Risk of All-Cause Mortality According to the Development of PaHF

**Figure 2:**
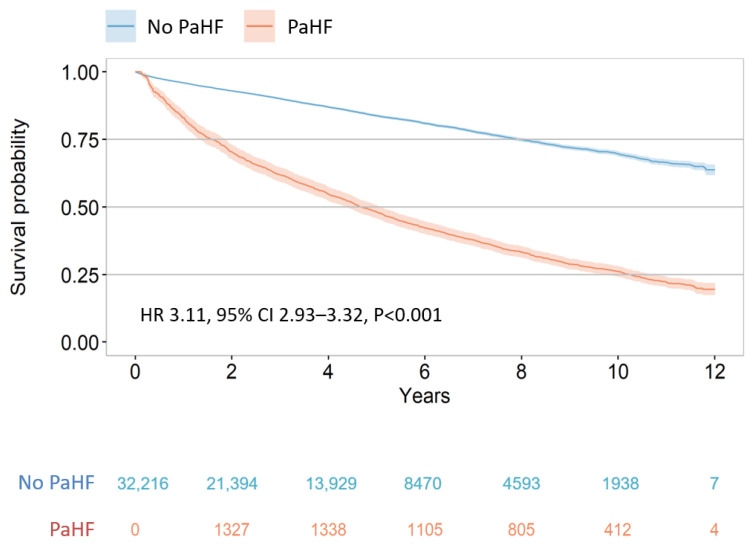
Extended Kaplan–Meier survival curves for all-cause death.

During the study period, all-cause deaths occurred in 6184 (19.2%) patients within the PPM cohort. Patients who developed PaHF exhibited a significantly higher incidence rate of death compared to those without PaHF (6.2 vs. 4.0 per 100 PYs; 95% CI, 5.9–6.5 vs. 3.9–4.1; *p* < 0.001). After accounting for immortal-time bias using extended Kaplan–Meier methods, PaHF remained consistently associated with worse survival outcomes (Figure 2). The 12-year cumulative survival probability was markedly lower in the PaHF group than in the non-PaHF group.

On multivariable Cox proportional hazards analysis (Model 1 of Table 2), PaHF development was independently associated with a more than threefold increased risk of all-cause mortality (HR, 3.11; 95% CI, 2.93–3.32; *p* < 0.001), even after adjusting for immortal-time bias and a broad range of clinical covariates including age, sex, comorbidities (DM, hypertension, coronary and peripheral artery disease, CKD/ESRD, valvular heart disease, AF, COPD), pacing indication, Charlson comorbidity index, pacemaker type (dual vs. single chamber), and use of HF medications (ACEIs/ARBs, beta-blockers, and MRAs).

**Figure 3:**
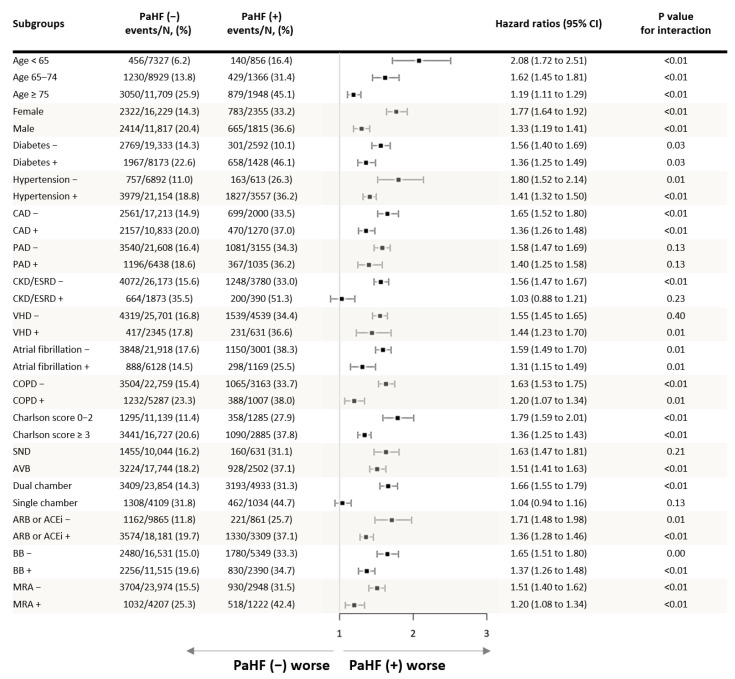
Subgroup analysis of the association between pacemaker-associated heart failure and all-cause mortality.

Subgroup analyses consistently demonstrated that PaHF development was associated with increased all-cause mortality across a wide range of clinical subgroups (Figure 3). The association between PaHF and mortality remained significant regardless of age, sex, or comorbidity burden. While the absolute incidence of PaHF was higher among older patients, males, and those with greater comorbidities, its relative impact on mortality appeared greater in younger patients, females, and those with fewer comorbidities.

**Figure 4:**
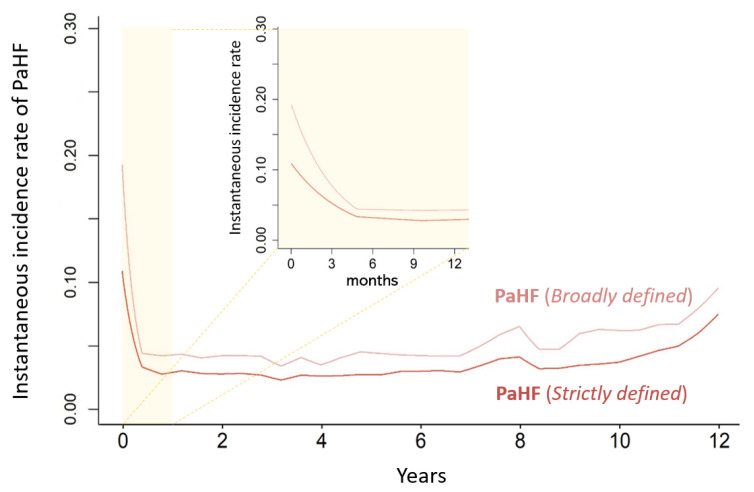
Instantaneous incidence rate of pacemaker-associated heart failure over time after permanent pacemaker implantation.

The instantaneous incidence rates of both strictly- and broadly defined PaHF peaked within the first 6 months after PPM implantation, followed by a gradual decline that stabilized but remained persistently above zero throughout the follow-up period (Figure 4). This pattern formed a characteristic L-shaped curve, reflecting an initial decline but a persistently elevated risk of PaHF throughout the follow-up period.

**Figure 5:**
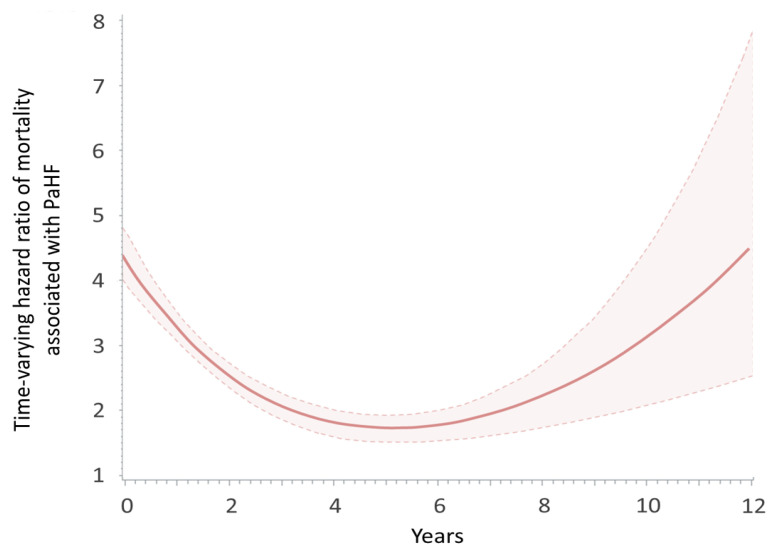
Time-varying hazard ratio of mortality associated with pacemaker-associated heart failure.

In the time-varying Cox model, the hazard ratio for PaHF-associated mortality was highest in the early post-implantation period and declined over time, reaching its lowest point around 5 years. However, the risk began to rise again thereafter, forming a U-shaped curve (Figure 5). Despite these temporal dynamics, PaHF consistently conferred a significantly elevated mortality risk across the entire follow-up duration.

### 3.3. Predictors of PaHF

Multivariable Cox regression analysis revealed several independent predictors of incident PaHF following de novo PPM implantation (Table 3). Older age—whether analyzed as a continuous or dichotomous variable (age ≥ 65 years)—was consistently associated with increased risk. In addition, male sex conferred a modest but statistically significant increase in risk. Among comorbidities, the presence of DM, hypertension, coronary artery disease, CKD/ESRD, valvular heart disease, AF, and chronic obstructive pulmonary disease were all independently associated with a higher likelihood of developing PaHF.

In contrast, a pacing indication of AVB as compared with SND and a history of peripheral artery disease were not significantly associated with PaHF risk after multivariable adjustment.

### 3.4. Sensitivity Analyses

Sensitivity analyses using the broad PaHF definition yielded results consistent with the primary analysis based on the strict definition. Patients with PaHF consistently showed significantly higher all-cause mortality than those without PaHF in the overall cohort (Supplementary Figures S3 and S4) as well as across various subgroups (Supplementary Figure S5). Additionally, extended multivariable models incorporating a non-parsimonious set of covariates produced similar results using both strict and broad definitions of PaHF (Table 3), with HRs ranging from 2.78 to 4.66.

## 4. Discussion

### 4.1. Main Findings

In this large-scale, nationwide cohort of patients with de novo PPM implantation, we found that: (1) PaHF developed in 12.9% and 19.0% of patients according to strict and broad definitions, respectively, during a median follow-up of 3.8 years; (2) PaHF was independently associated with a nearly threefold increased risk of all-cause mortality, even after accounting for immortal-time bias and potential confounders; and (3) both the incidence and mortality risk associated with PaHF were highest within the first 6 months post-implantation but persisted throughout long-term follow-up, with a notable resurgence in mortality risk approximately after five years.

These findings were derived from one of the largest unselected real-world PPM cohorts, using comprehensive national claims data encompassing nearly the entire Korean population, thus enhancing the generalizability of our results.

### 4.2. Incidence and Monitoring of PaHF

During a median follow-up of 3.8 years, PaHF occurred in 12.9% of patients when applying our strict definition, which likely captures moderate to severe forms of the condition. When the broader definition was used, the incidence rose to 19.0%, possibly due to the inclusion of milder cases in addition to more severe forms. These findings align with previous studies, including a single-center study reporting a 12.3% incidence using a similarly strict definition [2], and a U.S. MarketScan database study reporting a higher incidence of 25.8% when using broader diagnostic-code-based definitions [16].

Despite this significant incidence, there are no established guidelines regarding when or how often patients should be monitored post-PPM for PaHF. Previous studies have largely focused on the early follow-up. For instance, Tayal et al. suggested that echocardiographic evaluation at six-month would likely identify the majority of PaHF cases, as incidence was highest within the first six months post-PPM and declined thereafter [17]. However, their analysis did not extend beyond the early phase and lacked assessment of long-term risk. In contrast, our study demonstrated that PaHF risk, while highest in the initial 6 months, remains consistently elevated throughout long-term follow-up (Figure 4). These findings underscore the need for ongoing surveillance of LV function even beyond the early post-implantation period, especially in patients with predisposing clinical features.

### 4.3. Prognostic Impact of PaHF on Mortality

While the association between chronic RV pacing and incident HF has been well described, its impact on long-term mortality remains inconclusive, particularly among patients without preexisting HF [18]. Even in landmark randomized trials—such as DAVID, SAVE PACe, and DANPACE—a significant association between pacing burden and mortality was not observed [19,20,21]. Furthermore, pacing-minimization algorithms have consistently failed to reduce all-cause mortality in randomized controlled trials and meta-analyses [20,22,23]. One likely explanation is the relatively short follow-up durations in these studies, typically averaging 2.5 years or less, which may be insufficient to capture the delayed adverse effects of chronic RV pacing [4,19,20,22,23]. A recent Danish registry analysis compared the overall mortality of PPM recipients with that of the general population, offering broad insights into baseline risk associated with pacemaker implantation [17]. However, that study did not account for intermediate clinical events such as PaHF or model time-varying exposures, thereby limiting its ability to evaluate the downstream consequences of PaHF.

To address these limitations, we conducted a within-cohort comparison of mortality between patients who developed PaHF and those who did not, using a time-dependent Cox regression model in which PaHF was treated as a time-dependent covariate. By using a time-dependent approach, we captured how the timing of PaHF development influences long-term mortality outcomes. Additionally, with a follow-up duration exceeding four years, our study demonstrated that the development of PaHF was associated with a substantial and sustained increase in all-cause mortality. Notably, this risk followed a U-shaped trajectory—peaking early, reaching a nadir around five years, and resurging thereafter—suggesting that the long-term impact of pacing-induced LV dysfunction may not become fully apparent without extended observation. The late resurgence in mortality risk observed after approximately five years may reflect progressive ventricular remodeling due to chronic RV pacing, compounded by aging and accumulation of comorbidities. Additional contributors such as worsening renal function, atrial fibrillation progression, or valvular dysfunction may also play a role. While speculative, these findings underscore that PaHF represents a dynamic and evolving risk, highlighting the importance of continued long-term surveillance beyond the early post-implantation phase.

By explicitly modeling time-varying exposure and conducting multiple sensitivity analyses, our study provides robust evidence that PaHF is a key determinant of adverse outcomes following PPM implantation. These findings highlight the importance of long-term surveillance and suggest that early identification and intervention—such as the use of conduction system pacing (CSP) in high-risk individuals—may help mitigate downstream mortality risk.

### 4.4. Risk Factors of PaHF

Consistent with prior literature, we identified advanced age (≥65 years), male sex, DM, CKD/ESRD, and AF as independent predictors of PaHF [4,5,6,16,17,24]. However, contrary to our initial expectation, AVB was not associated with an increased risk of PaHF (Table 2). This finding may be explained by the fact that pacing indication alone does not accurately reflect actual RV pacing burden. For example, in the DANPACE study, patients with SND had an unexpectedly high RV pacing burden (~85%), despite being presumed at low risk, when treated with dual-chamber PPM [25]. In contrast, AVB patients in the IDEAL RVP trial maintained RV pacing below 40% when algorithms to minimize pacing were used [26]. Similarly, a German registry found no difference in PaHF incidence or mortality between AVB and SND groups [4]. These findings caution against using pacing indication alone as a surrogate for RV pacing burden.

### 4.5. Prevention Strategies and Subgroup Considerations

Given the relatively modest incidence of PaHF (10–25%) observed in our data and most recent large-scale registry-based studies, prophylactic CRT implantation in patients with preserved LV function or no prior HF may not be routinely warranted [16,17]. Rather, alternative strategies such as CSP may be more appropriate, especially in patients at higher risk for PaHF. Our findings suggest that younger patients may derive greater benefit from such physiologic pacing approaches [7,27], as the relative impact of PaHF on mortality was more pronounced in younger age groups, despite higher absolute mortality observed among older individuals (Figure 3). This contrast likely reflects competing mortality risks in elder patients, where comorbidities may overshadow the prognostic effects of RV pacing. In contrast, younger patients, with longer anticipated pacing exposure and fewer competing risks, may be disproportionately affected by pacing-induced dyssynchrony. A similar trend was seen with comorbidity status: although patients with a greater comorbidity burden were more likely to develop PaHF, their prognosis may have been more influenced by underlying conditions. In contrast, patients with fewer competing risks—such as the young and otherwise healthy—had a lower incidence of PaHF, but its development appeared to have a more pronounced impact on their long-term prognosis. Collectively, these subgroup findings support the importance of individualized pacing strategies based on patient-specific risk profiles.

### 4.6. Implications in the Era of New Conduction System and Leadless Pacing

Although CSP offers promising physiologic benefits and leadless pacemakers are increasingly used for their low complication rates, conventional RV pacing remains widely practiced [28]. Importantly, leadless systems still induce ventricular dyssynchrony and do not eliminate the risk of PaHF [29,30]. In this context, our findings provide timely and clinically relevant benchmarks on the long-term risks of conventional RV pacing. These results may guide future evaluation of newer pacing modalities, including CSP and leadless systems, by offering a reference point for comparison. Furthermore, our findings highlight an unmet need to define optimal management and surveillance strategies for patients who develop PaHF. Although our study was not designed to evaluate specific interventions, future prospective studies should determine the clinical impact and optimal timing of CRT upgrade, conversion to CSP, or intensification of guideline-directed medical therapy. In addition, the most effective surveillance intervals and modalities—such as echocardiography or biomarker monitoring—require clarification, particularly in high-risk subgroups.

### 4.7. Limitations

We acknowledge several limitations. First, echocardiographic data were not available in this nationwide claims-based dataset. As a result, we could not distinguish the incidence of PaHF according to LV EF (i.e., preserved vs. reduced). Nevertheless, our sensitivity analysis using a broader PaHF definition—likely including cases with preserved EF—yielded results consistent with the primary findings. Second, device-related data, including RV-pacing percentage or site (apex vs. septum) and use of RV-pacing minimization algorithms, were not accessible. This precluded a quantitative assessment of the relationship between pacing burden, pacing site, algorithm use and the development or prognosis of PaHF. The absence of RV-pacing data also limits our ability to definitively attribute PaHF cases to RV-pacing-induced electromechanical dyssynchrony. Future prospective studies incorporating detailed echocardiographic, device interrogation, and imaging data are needed to refine risk stratification, establish pacing burden thresholds, and evaluate the role of specific pacing algorithms or pacing site in mitigating long-term adverse outcomes. Third, our study assumed that incident HF occurring after pacemaker implantation was pacing-associated. However, HF can also arise independently from aging and multiple comorbidities. While our design excluded prior HF and censored for several non-pacing HF etiologies, we were unable to directly compare our findings with a non-paced general population. Therefore, the incidence of PaHF reported in this study may be overestimated, and causal inference should be interpreted with caution. Fourth, given the potential analytical complexity and risk of overextending the current study scope, we did not evaluate the therapeutic impact of pacing modality changes, such as CRT-upgrade or transition to CSP, in patients who developed PaHF. This important question will be explored in a separate investigation. Finally, our study was conducted in a Korean population within a single-payer healthcare system. While the pathophysiology of pacing-induced dyssynchrony is universal, differences in demographics and healthcare delivery may affect absolute event rates. Thus, external validation in other populations and healthcare systems is warranted. Despite these limitations, our study provides robust real-world evidence on the incidence and long-term prognostic impact of PaHF following de novo PPM implantation, offering important insights for future risk stratification and pacing strategy optimization.

## 5. Conclusions

In this large, nationwide real-world cohort, the development of PaHF was independently associated with a substantial and sustained increase in all-cause mortality following de novo PPM implantation. Both the incidence of PaHF and the associated mortality risk were highest within the first six months post implantation; however, our findings also revealed that these risks did not dissipate over time but instead persisted—and in the case of mortality, re-emerged—during long-term follow-up.

These time-dependent patterns highlight PaHF as a dynamic and clinically meaningful complication of conventional RV pacing. Given the ongoing risk beyond the early post-implantation phase, routine and longitudinal monitoring of cardiac function may be warranted. Our findings may inform the development of tailored pacing strategies and surveillance protocols aimed at mitigating long-term adverse outcomes in this growing patient population.

## Figures and Tables

**Table 1:** Baseline characteristics of the permanent pacemaker cohort.

Variables	Overall (n = 32,216)	PaHF (n = 4170)	No PaHF (n = 28,046)	aSMD	*p* Value
Demographics and medical history					
Age, years	70.6 ± 12.1	71.9 ± 11.2	70.4 ± 12.2	0.128	<0.001
Age ≥ 65 years	24,033 (74.6)	3314 (79.5)	20,719 (73.9)	0.133	<0.001
Male	13,632 (42.3)	1815 (43.5)	11,817 (42.1)	0.028	0.093
Diabetes	10,291 (31.9)	1578 (37.8)	8713 (31.1)	0.143	<0.001
Hypertension	24,711 (76.7)	3557 (85.3)	21,154 (75.4)	0.250	<0.001
Coronary artery disease	13,003 (40.4)	2170 (52.0)	10,833 (38.6)	0.272	<0.001
Peripheral artery disease	7453 (23.1)	1015 (24.3)	6438 (23.0)	0.033	0.050
CKD/ESRD	2263 (7.0)	390 (9.4)	1873 (6.7)	0.099	<0.001
Valvular heart disease	2976 (9.2)	631 (15.1)	2345 (8.4)	0.211	<0.001
Atrial fibrillation	7297 (22.7)	1169 (28.0)	6128 (21.8)	0.143	<0.001
COPD	6294 (19.5)	1007 (24.1)	5287 (18.9)	0.129	<0.001
CCI ≥ 3 unit	19,612 (60.9)	2885 (69.2)	16,727 (59.6)	0.200	<0.001
Pacemaker-related variables					
AV block	20,246 (63.4)	2502 (60.0)	17,744 (63.3)	0.068	<0.001
Sinus node dysfunction	11,675 (36.6)	1631 (39.1)	10,044 (35.8)		
Dual chamber	27,073 (84.0)	3136 (75.2)	23,937 (85.3)	0.262	<0.001
Medications					
ACEIs or ARBs	21,490 (66.7)	3309 (79.4)	18,181 (64.8)	0.328	<0.001
Beta blockers	13,905 (43.2)	2390 (57.3)	11,515 (41.1)	0.330	<0.001
MRAs	5274 (16.4)	1222 (29.3)	4072 (14.5)	0.363	<0.001
Loop diuretics	12,611 (39.1)	2389 (57.3)	10,222 (36.4)	0.427	<0.001
Thiazide	6200 (19.2)	1038 (24.9)	5162 (18.4)	0.158	<0.001
Antiplatelet agents	19,301 (59.9)	2990 (71.7)	16,311 (58.2)	0.287	<0.001
Anticoagulants	6382 (19.8)	1127 (27.0)	5255 (18.7)	0.198	<0.001

Values are expressed as means ± standard deviations or n (%). Abbreviations: PaHF, pacemaker-associated heart failure; aSMD, absolute standardised mean difference; CKD, chronic kidney disease; ESRD, end-stage renal disease; COPD, chronic obstructive pulmonary disease; CCI, Charlson comorbidity index; AV block, atrioventricular block; ACEIs, angiotensin-converting enzyme inhibitors; ARBs, angiotensin II receptor antagonists; MRAs, mineralocorticoid receptor antagonists.

**Table 2:** All-cause mortality risk of strictly- and broadly defined pacemaker-associated heart failure in the permanent pacemaker cohort.

	PaHF (Strictly Defined)			PaHF (Broadly Defined)		
	HR	95% CI	*p*-Value	HR	95% CI	*p*-Value
Unadjusted analysis	3.87	3.64–4.12	<0.001	5.90	5.59–6.23	<0.001
Multivariable analysis model 1 *	3.11	2.93–3.32	<0.001	4.85	4.59–5.13	<0.001
Multivariable analysis model 2 *	2.92	2.74–3.11	<0.001	4.39	4.16–4.65	<0.001
Multivariable analysis model 3 *	2.78	2.61–2.96	<0.001	4.25	4.02–4.50	<0.001
Multivariable analysis model 4 *	2.95	2.77–3.14	<0.001	4.66	4.41–4.93	<0.001

*, Multivariable analysis models 1 and 2 were adjusted for variables with a *p* value < 0.05 in the univariable analysis such as age (as a dichotomous variable of ‘≥65 years’ in model 1 while as a continuous variable in model 2), sex, diabetes mellitus, hypertension, coronary artery disease, peripheral artery disease, CKD/ESRD, valvular heart disease, atrial fibrillation, COPD, and pacing indication. Multivariable analysis models 3 and 4 incorporated variables in a non-parsimonious manner, including the variables in the Models 1 and 2, respectively, and additional variables such as dual (vs. single) chamber PPM, ACEIs or ARBs, beta blockers, and MRAs.

**Table 3:** Independent risk factors of pacemaker-associated heart failure in the permanent pacemaker cohort.

Variable	Univariable Analyses		Multivariable Analysis Model 1		Multivariable Analysis Model 2	
	HR (95% CI)	*p* Value	HR (95% CI)	*p* Value	HR (95% CI)	*p* Value
Age (continuous)	1.03 (1.02–1.03)	<0.001	1.02 (1.02–1.02)	<0.001		
Age ≥ 65 years	1.77 (1.64–1.91)	<0.001			1.43 (1.32–1.55)	<0.001
Male	1.15 (1.08–1.22)	<0.001	1.09 (1.03–1.16)	0.005	1.08 (1.01–1.15)	0.023
Diabetes mellitus	1.53 (1.44–1.63)	<0.001	1.15 (1.07–1.23)	<0.001	1.14 (1.06–1.22)	<0.001
Hypertension	2.06 (1.90–2.25)	<0.001	1.41 (1.29–1.54)	<0.001	1.49 (1.36–1.64)	<0.001
CAD	1.66 (1.56–1.77)	<0.001	1.35 (1.26–1.43)	<0.001	1.36 (1.28–1.45)	<0.001
PAD	1.19 (1.11–1.27)	<0.001	0.97 (0.90–1.04)	0.355	0.97 (0.90–1.05)	0.471
CKD/ESRD	2.13 (1.92–2.37)	<0.001	1.48 (1.32–1.65)	<0.001	1.49 (1.34–1.67)	<0.001
Valvular heart disease	1.80 (1.65–1.96)	<0.001	1.69 (1.55–1.84)	<0.001	1.65 (1.51–1.80)	<0.001
Atrial fibrillation	1.92 (1.79–2.06)	<0.001	1.70 (1.58–1.83)	<0.001	1.70 (1.58–1.84)	<0.001
COPD	1.44 (1.34–1.54)	<0.001	1.18 (1.10–1.27)	<0.001	1.20 (1.11–1.29)	<0.001
AVB versus SND	0.90 (0.85–0.96)	0.001	0.99 (0.92–1.05)	0.679	1.00 (0.94–1.07)	0.985

## Data Availability

This study utilized publicly available data that can be accessed only after obtaining official approval.

## Supplementary Materials

The following supporting information can be downloaded at: https://www.mdpi.com/article/10.3390/jcm14217744/s1, Table S1: Cardiac implantable electronic device and procedure definitions. Table S2: Variable definitions by International Classification of diseases, 10th revision (ICD-10) codes. Table S3: Comorbidity definitions for Charlson comorbidity index using International Classification of Diseases, 10th revision (ICD-10) codes. Table S4: Details of the PaHF definition. Table S5: HF-related causes of censoring for PaHF incidence outcome. Figure S1: Graphical depiction of immortal-time bias in the PPM cohort. Figure S2: Cumulative incidence rates of PaHF development according to strict and broad definitions. Figure S3: Extended Kaplan—Meier survival curves for all-cause death in PPM cohort according to the occurrence of broadly defined PaHF. Figure S4: Time-varying hazard ratio of mortality associated with broadly defined PaHF throughout entire follow-up period. Figure S5: Forest plot of association between broadly defined PaHF and all-cause mortality by subgroups.

## Abbreviations

ACEI	angiotensin-converting enzyme inhibitor
AF	atrial fibrillation
ARB	angiotensin receptor blocker
AVB	atrioventricular block
CAD	coronary artery disease
CCI	Charlson comorbidity index
COPD	chronic obstructive pulmonary disease
CKD	chronic kidney disease
CRT	cardiac resynchronization therapy.
ESRD	end-stage renal disease
MRA	mineralocorticoid receptor antagonist
PAD	peripheral artery disease
PaHF	pacemaker-associated heart failure
PPM	permanent pacemaker
SND	sinus node dysfunction
VHD	valvular heart disease
