# Supplementary material for: Temporal Trends and Prognostic Impact of Pacemaker-Associated Heart Failure: Insights from a Nationwide Cohort Study
Source: J Clin Med. 2025 Oct 31;14(21):7744. doi: 10.3390/jcm14217744 (PMC12610581; doi:10.3390/jcm14217744)
Supplement: Supplementary file 1 [file jcm-14-07744-s001.zip › jcm-3950933-supplementary.pdf]

Journal of Clinical Medicine

Supplementary Table S1. Cardiac implantable electronic device and procedure definitions.

Variable	Procedure Codes	Device Codes
**Pacemaker implantation**		
Single chamber	O0203	G8201, G8202
Dual chamber	O0204	G8203, G8204, G8205
**ICD implantation**		
Single chamber	O0211	G8301
Dual chamber	O0211	G8302
**CRT implantation**		
CRT-P	O0203 or O0204	G8103
CRT-D	O0211	G8303

Supplementary Table S2. Variable definitions by International Classification of diseases, 10th revision (ICD-10) codes

Variable	ICD-10	Diagnostic Definition
Diabetes	E11-E14	Admission≥1 or outpatient department ≥ 2
Hypertension	I10-I13, I15	Admission≥1 or outpatient department ≥ 2
Coronary artery disease	I20-I25	Admission≥1 or outpatient department ≥ 2
Peripheral artery disease	I70, I73	Admission or outpatient department ≥ 2
CKD/ESRD	I13.1, N03, N05, N10-N19, Z49, Z94.0, Z99.2	Admission or outpatient department ≥ 1
Valvular heart disease	I05, I06, I07, I08, I34, I35, I36, I37, I39	Admission≥1 or outpatient department ≥ 2
Atrial fibrillation	I48.0-48.4, I48.9	Admission≥1 or outpatient department ≥ 2
COPD	J41-44	Admission or outpatient department ≥ 1
AV block	I441, I442, I443	
Sinus node dysfunction	I495	

Supplementary Table S3. Comorbidity definitions for Charlson comorbidity index using International Classification of Diseases, 10th revision (ICD-10) codes

Variable	ICD-10
Myocardial infarction	I21.x, I22.x, I25.2
Congestive heart failure	I09.9, I11.0, I13.0, I13.2, I25.5, I42.0, I42.5 - I42.9, I43.x, I50.x, P29.0
Peripheral vascular disease	I70.x, I71.x, I73.1, I73.8, I73.9, I77.1, I79.0, I79.2, K55.1, K55.8, K55.9, Z95.8, Z95.9
Cerebrovascular disease	G45.x, G46.x, H34.0, I60.x - I69.x
Dementia	F00.x - F03.x, F05.1, G30.x, G31.1
Chronic pulmonary disease	I27.8, I27.9, J40.x - J47.x, J60.x - J67.x, J68.4, J70.1, J70.3
Rheumatic disease	M05.x, M06.x, M31.5, M32.x - M34.x, M35.1, M35.3, M36.0
Peptic ulcer disease	K25.x - K28.x
Mild liver disease	B18.x, K70.0 - K70.3, K70.9, K71.3 - K71.5, K71.7, K73.x, K74.x, K76.0, K76.2 - K76.4, K76.8, K76.9, Z94.4
Diabetes without chronic complication	E10.0, E10.1, E10.6, E10.8, E10.9, E11.0, E11.1, E11.6, E11.8, E11.9, E12.0, E12.1, E12.6, E12.8, E12.9, E13.0, E13.1, E13.6, E13.8, E13.9, E14.0, E14.1, E14.6, E14.8, E14.9
Diabetes with chronic complication	E10.2 - E10.5, E10.7, E11.2 - E11.5, E11.7, E12.2 - E12.5, E12.7, E13.2 - E13.5, E13.7, E14.2 - E14.5, E14.7
Hemiplegia or paraplegia	G04.1, G11.4, G80.1, G80.2, G81.x, G82.x, G83.0 - G83.4, G83.9
Renal disease	I12.0, I13.1, N03.2 - N03.7, N05.2 - N05.7, N18.x, N19.x, N25.0, Z49.0 - Z49.2, Z94.0, Z99.2

Any malignancy, including lymphoma and leukemia, except malignant skin cancer	C00.x - C26.x, C30.x - C34.x, C37.x - C41.x, C43.x, C45.x - C58.x, C60.x - C76.x, C81.x - C85.x, C88.x, C90.x - C97.x
Moderate or severe liver disease	I85.0, I85.9, I86.4, I98.2, K70.4, K71.1, K72.1, K72.9, K76.5, K76.6, K76.7
Metastatic solid tumor	C77.x - C80.x
AIDS/HIV	B20.x - B22.x, B24.x

Supplementary Table S4. Details of the PaHF definition

Strict PaHF Definition [Any of 1), 2), 3)]	Broad PaHF Definition [Any of 1), 2), 3)]
Different criteria	
(1) Hospitalization with newly assigned HF code (I50.9) and ≥2 claims for HF medications among ACEIs/ARBs, beta-blockers, or MRAs	(1) Hospitalization with newly assigned HF code (I50.9)
Common criteria	
(2) newly prescribed ARNI therapy	(2) newly prescribed ARNI therapy
(3) upgrade to CRT device	(3) upgrade to CRT device

Supplementary Table S5. HF-related causes of censoring for PaHF-incidence outcome

Disease	Code
Myocardial infarction, hospitalised	I21 (ICD-10) with hospitalization
Myocardial infarction treated with PCI‡	I21, I22, I23 (ICD-10) + coronary angiography (procedure code: HA670)
Myocarditis, hospitalised cases	I01.2, I09.0, I40.0–40.9, I41.0–41.8, I51.4 (ICD-10)
Alcoholic cardiomyopathy	425.5 (ICD-9) or I42.6 (ICD-10)
Cardiac Sarcoidosis	V111 & D860-D863, D868, D869 (ICD-10)
Cardiac amyloidosis (TTR, AL)	E85 (ICD-10)

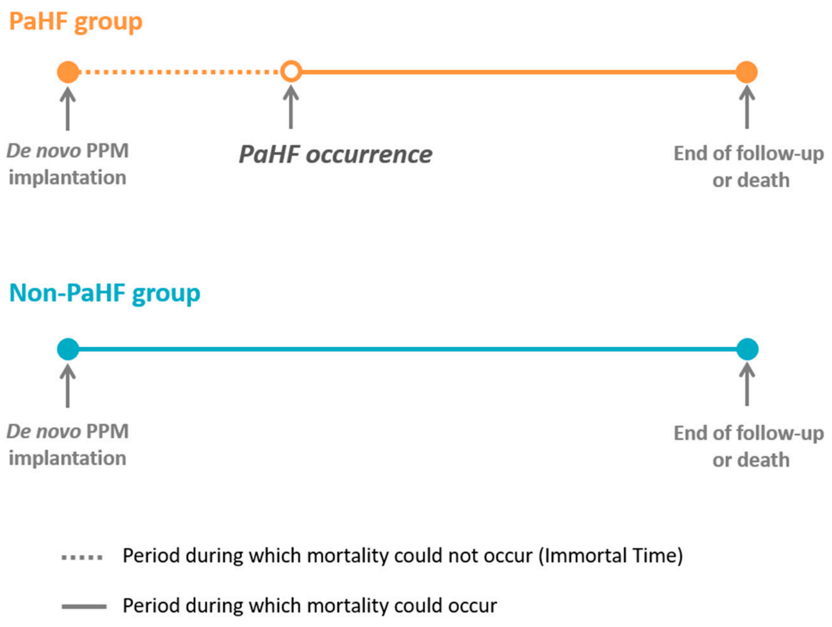

Supplementary Figure S1. Graphical depiction of immortal-time bias in the PPM cohort.

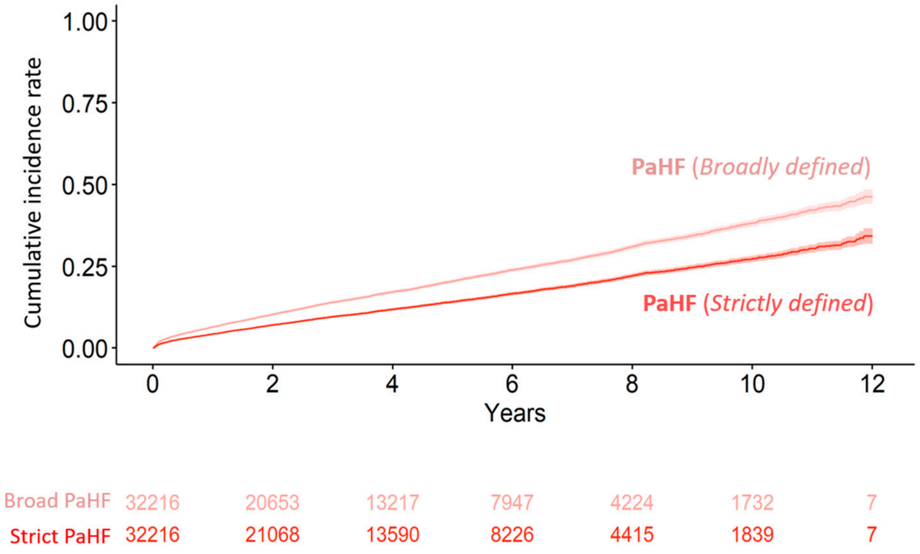

Supplementary Figure S2. Cumulative incidence rates of PaHF development according to strict and broad definitions.

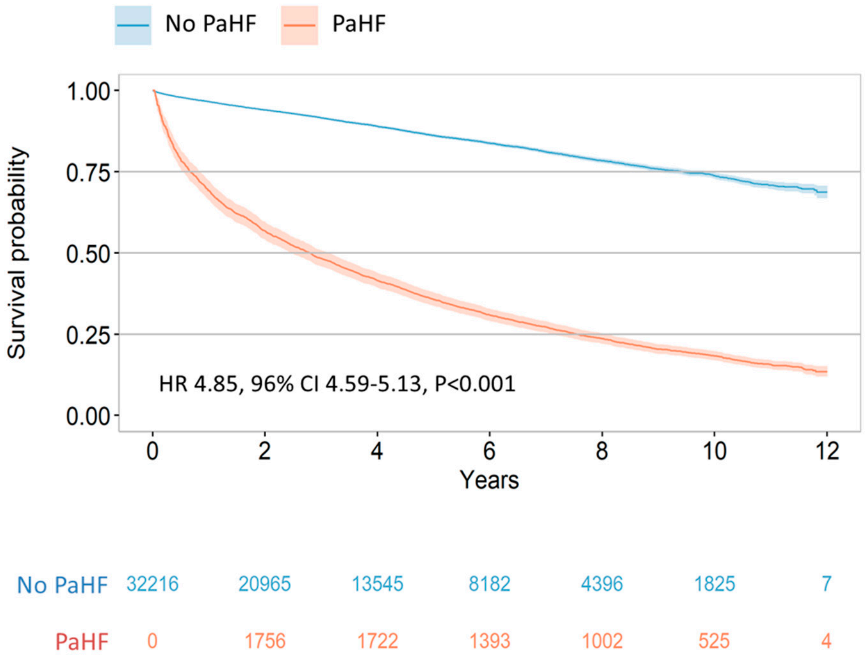

Supplementary Figure S3. Extended Kaplan–Meier survival curves for all-cause death in PPM cohort according to the occurrence of broadly-defined PaHF.

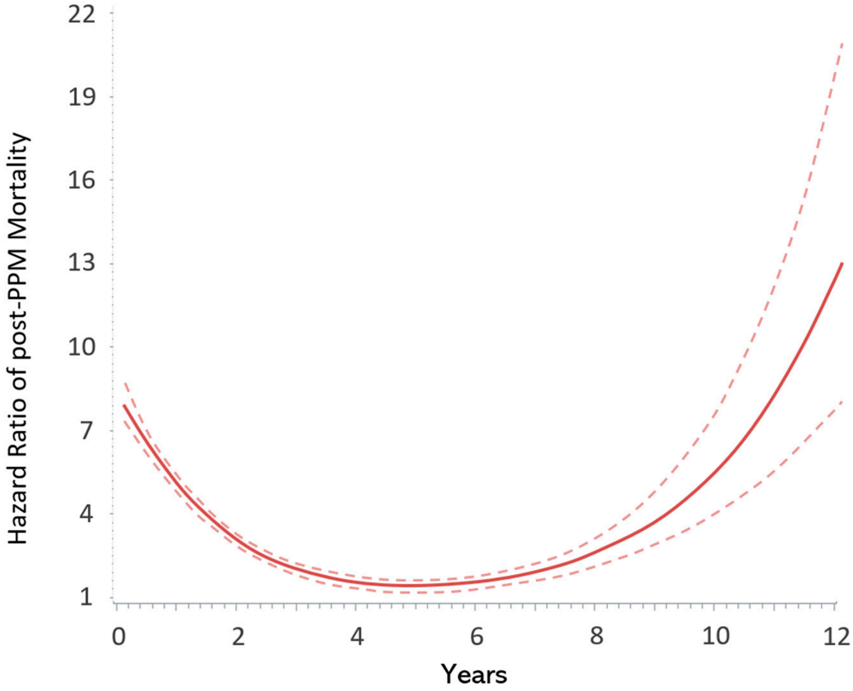

Supplementary Figure S4. Time-varying hazard ratio of mortality associated with broadly-defined PaHF throughout entire follow-up period.

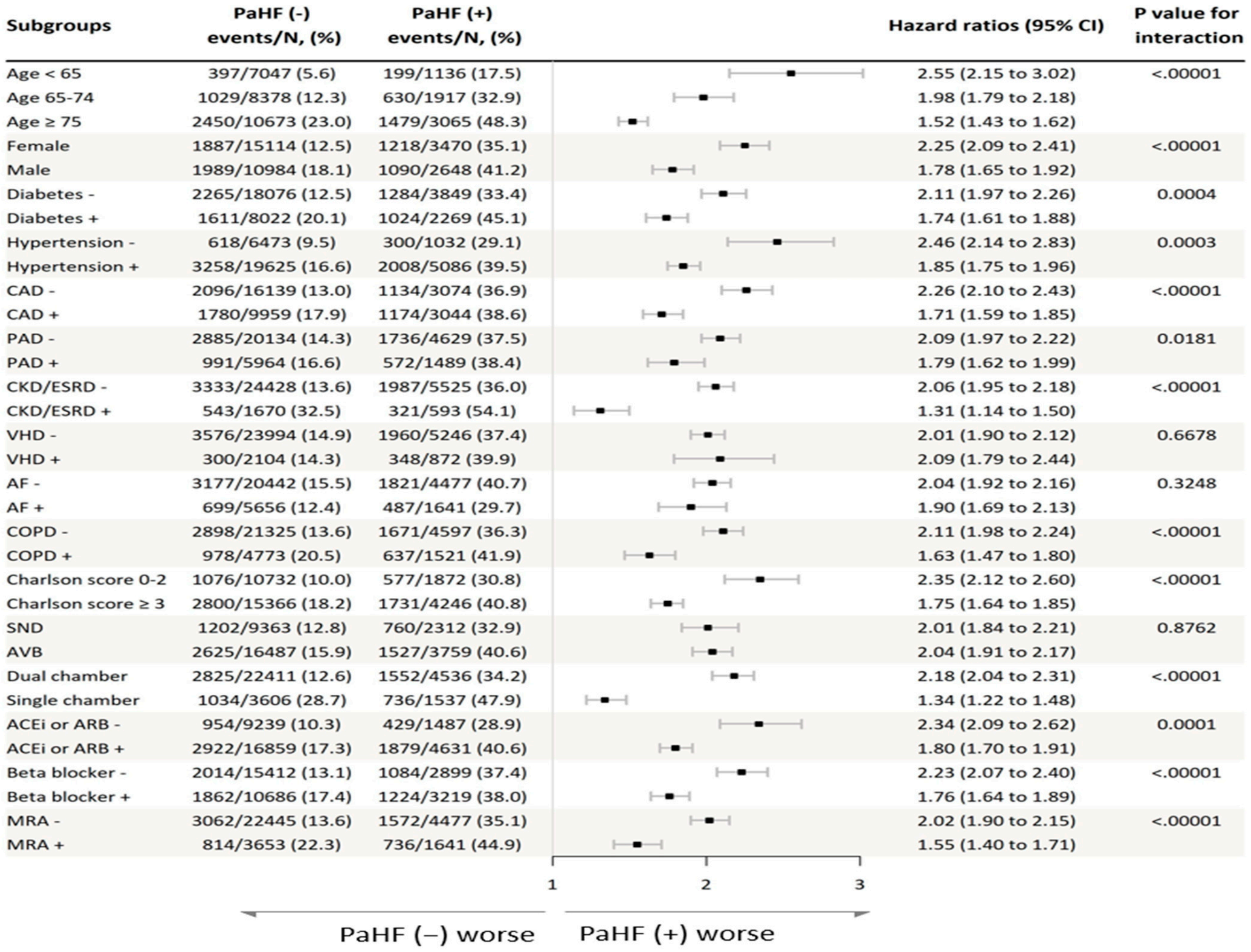

Subgroups	PaHF (-) events/N, (%)	PaHF (+) events/N, (%)	Hazard ratios (95% CI)	P value for interaction
Age < 65	397/7047 (5.6)	199/1136 (17.5)	2.55 (2.15 to 3.02)	<.00001
Age 65-74	1029/8378 (12.3)	630/1917 (32.9)	1.98 (1.79 to 2.18)	
Age ≥ 75	2450/10673 (23.0)	1479/3065 (48.3)	1.52 (1.43 to 1.62)	
Female	1887/15114 (12.5)	1218/3470 (35.1)	2.25 (2.09 to 2.41)	<.00001
Male	1989/10984 (18.1)	1090/2648 (41.2)	1.78 (1.65 to 1.92)	
Diabetes -	2265/18076 (12.5)	1284/3849 (33.4)	2.11 (1.97 to 2.26)	0.0004
Diabetes +	1611/8022 (20.1)	1024/2269 (45.1)	1.74 (1.61 to 1.88)	
Hypertension -	618/6473 (9.5)	300/1032 (29.1)	2.46 (2.14 to 2.83)	0.0003
Hypertension +	3258/19625 (16.6)	2008/5086 (39.5)	1.85 (1.75 to 1.96)	
CAD -	2096/16139 (13.0)	1134/3074 (36.9)	2.26 (2.10 to 2.43)	<.00001
CAD +	1780/9959 (17.9)	1174/3044 (38.6)	1.71 (1.59 to 1.85)	
PAD -	2885/20134 (14.3)	1736/4629 (37.5)	2.09 (1.97 to 2.22)	0.0181
PAD +	991/5964 (16.6)	572/1489 (38.4)	1.79 (1.62 to 1.99)	
CKD/ESRD -	3333/24428 (13.6)	1987/5525 (36.0)	2.06 (1.95 to 2.18)	<.00001
CKD/ESRD +	543/1670 (32.5)	321/593 (54.1)	1.31 (1.14 to 1.50)	
VHD -	3576/23994 (14.9)	1960/5246 (37.4)	2.01 (1.90 to 2.12)	0.6678
VHD +	300/2104 (14.3)	348/872 (39.9)	2.09 (1.79 to 2.44)	
AF -	3177/20442 (15.5)	1821/4477 (40.7)	2.04 (1.92 to 2.16)	0.3248
AF +	699/5656 (12.4)	487/1641 (29.7)	1.90 (1.69 to 2.13)	
COPD -	2898/21325 (13.6)	1671/4597 (36.3)	2.11 (1.98 to 2.24)	<.00001
COPD +	978/4773 (20.5)	637/1521 (41.9)	1.63 (1.47 to 1.80)	
Charlson score 0-2	1076/10732 (10.0)	577/1872 (30.8)	2.35 (2.12 to 2.60)	<.00001
Charlson score ≥ 3	2800/15366 (18.2)	1731/4246 (40.8)	1.75 (1.64 to 1.85)	
SND	1202/9363 (12.8)	760/2312 (32.9)	2.01 (1.84 to 2.21)	0.8762
AVB	2625/16487 (15.9)	1527/3759 (40.6)	2.04 (1.91 to 2.17)	
Dual chamber	2825/22411 (12.6)	1552/4536 (34.2)	2.18 (2.04 to 2.31)	<.00001
Single chamber	1034/3606 (28.7)	736/1537 (47.9)	1.34 (1.22 to 1.48)	
ACEi or ARB -	954/9239 (10.3)	429/1487 (28.9)	2.34 (2.09 to 2.62)	0.0001
ACEi or ARB +	2922/16859 (17.3)	1879/4631 (40.6)	1.80 (1.70 to 1.91)	
Beta blocker -	2014/15412 (13.1)	1084/2899 (37.4)	2.23 (2.07 to 2.40)	<.00001
Beta blocker +	1862/10686 (17.4)	1224/3219 (38.0)	1.76 (1.64 to 1.89)	
MRA -	3062/22445 (13.6)	1572/4477 (35.1)	2.02 (1.90 to 2.15)	<.00001
MRA +	814/3653 (22.3)	736/1641 (44.9)	1.55 (1.40 to 1.71)	

Supplementary Figure S5. Forest plot of association between broadly-defined PaHF and all-cause mortality by subgroups.
